# Growth Performance, Meat Quality and Antioxidant Status of Sheep Supplemented with Tannins: A Meta-Analysis

**DOI:** 10.3390/ani11113184

**Published:** 2021-11-08

**Authors:** José Felipe Orzuna-Orzuna, Griselda Dorantes-Iturbide, Alejandro Lara-Bueno, Germán David Mendoza-Martínez, Luis Alberto Miranda-Romero, Héctor Aarón Lee-Rangel

**Affiliations:** 1 Departamento de Zootecnia, Universidad Autónoma Chapingo, Chapingo CP 56230, Mexico; jforzuna@gmail.com (J.F.O.-O.); griseldi0993@gmail.com (G.D.-I.); albertomiranda@correo.chapingo.mx (L.A.M.-R.); 2 Unidad Xochimilco, Departamento de Producción Agrícola y Animal, Universidad Autónoma Metropolitana, Mexico City CP 04960, Mexico; gmendoza@correo.xoc.uam.mx; 3 Centro de Biociencias, Facultad de Agronomía y Veterinaria, Instituto de Investigaciones en Zonas Desérticas, Universidad Autónoma de San Luis Potosí, San Luis Potosí CP 78321, Mexico; hector.lee@uaslp.mx

**Keywords:** oxidative stability, natural antioxidants, polyphenolic compounds, meta-regression

## Abstract

**Simple Summary:**

Tannins can be used to improve productive performance, meat quality and antioxidant status of ruminants. The objective of this study was to evaluate the effects of dietary tannin supplementation on productive performance, carcass characteristics, meat quality and blood serum antioxidant status of sheep through a meta-analysis. Only studies with weaned or older sheep were included. The sheep included in the present study were between 2 and 6 months old, and between 12 and 31 kg of body weight. Tannin supplementation improved productive performance, carcass yield, meat oxidative stability and blood serum antioxidant capacity. This suggests that the inclusion of tannins in sheep diets could be used to improve growth and reduce oxidative stress in animals, and to improve meat quality and shelf life.

**Abstract:**

The objective of this study was to evaluate the effects of dietary supplementation with tannins (TANs) on productive performance, carcass characteristics, meat quality, oxidative stability, and blood serum antioxidant capacity of sheep through a meta-analysis. Using Scopus, Web of Science, ScienceDirect, and PubMed databases, a systematic search was performed for studies published in scientific journals that investigated the effects of TANs supplementation on the variables of interest. Only studies with weaned or older sheep were included. The data analyzed were extracted from 53 peer-reviewed publications. The sheep included in the present study were between 2 and 6 months old, and between 12 and 31 kg of body weight. The effects of TANs were analyzed using random-effects statistical models to examine the standardized mean difference (SMD) between treatments with TANs and control (no TANs). Heterogeneity was explored by meta-regression and a subgroup analysis was performed for covariates that were significant. Supplementation with TANs did not affect dry matter intake, pH, color (L* and b*), Warner–Bratzler shear force, cooking loss and meat chemical composition (*p* > 0.05). Supplementation with TANs increased daily weight gain (SMD = 0.274, *p* < 0.05), total antioxidant capacity (SMD = 1.120, *p* < 0.001), glutathione peroxidase enzyme activity (SMD = 0.801, *p* < 0.001) and catalase (SMD = 0.848, *p* < 0.001), and decreased malondialdehyde (MDA) concentration in blood serum (SMD = −0.535, *p* < 0.05). Supplementation with TANs decreased feed conversion rate (SMD = −0.246, *p* < 0.05), and the concentration of MDA (SMD = −2.020, *p* < 0.001) and metmyoglobin (SMD = −0.482, *p* < 0.05) in meat. However, meat redness (SMD = 0.365), hot carcass yield (SMD = 0.234), cold carcass yield (SMD = 0.510), backfat thickness (SMD = 0.565) and the *Longissimus dorsi* muscle area (SMD = 0.413) increased in response to TANs supplementation (*p* < 0.05). In conclusion, the addition of tannins in sheep diets improves productive performance, antioxidant status in blood serum, oxidative stability of meat and some other characteristics related to meat and carcass quality.

## 1. Introduction

Antibiotics (e.g., monensin) have been used for several decades as growth promoters in animals [1]. However, the inappropriate use of these products results in an accumulation of toxic residues in meat, which can affect the health of the consumer [2,3]. In addition, the emergence of bacterial strains resistant to the antibiotic effects [4] as well as the prohibition of these compounds in some countries [5] have led the industry and researchers to search for alternative products with similar effects as antibiotics, but of natural origin. Tannins (TANs), which are derived from plants, have received special attention and are among the most studied bioactive compounds, particularly in ruminants [1]. TANs are a group of polyphenolic compounds present in a wide variety of plants, which can be grouped into hydrolysable tannins (HTs) and condensed tannins (CTs) based on their chemical structure [6,7]. TANs can produce positive effects in animals, such as those of the antioxidant, antimicrobial, antiparasitic, immunomodulatory, and anti-inflammatory varities [1,6].

TANs can play an important role in the nutritional value of the feed, the quality of the products obtained, and the health and welfare of the animals [8]. Dietary inclusion of TANs at low to moderate concentrations (20 to 45 g kg^−1^ DM) can improve growth rate and feed utilization efficiency in ruminants, mainly due to a reduction in protein degradation in the rumen and a subsequent increase in the flow of amino acids to the small intestine [9,10,11]. However, large amounts (>55 g kg^−1^ DM) of TANs in the diet reduce feed intake, rumen microbial activity, nutrient digestibility and endogenous digestive enzyme activity [1,9,10,12], resulting in lower feed efficiency and growth rate.

Particularly in sheep, several studies have been conducted to evaluate the effects of dietary supplementation with extracts of TANs and TANs-rich plants on productive performance [13,14], carcass characteristics [15,16], oxidative stability and physicochemical characteristics of meat [17,18,19,20], and blood serum antioxidant status [21,22]. However, the results are still not consistent, probably as a consequence of variability among the studies regarding feeding conditions, age of the animals, type of product used, dosage and source of TANs [1,8]. Therefore, identifying the factors that contribute to this variability is a key aspect in the development of products containing TANs that can be used to improve productive performance, meat and carcass quality, and antioxidant status of sheep.

Some review articles [1,8,23,24] have suggested that dietary supplementation with TANs can improve productive performance, meat quality and antioxidant status of livestock. However, these reviews did not use a meta-analysis approach and also did not focus only on sheep. In addition, narrative reviews can lead to biased conclusions because they lack a methodological approach and are subjective to the author’s interpretation of previous research [25]. In contrast, meta-analysis (MA) is a statistical tool that allows synthesizing data published in different studies in a quantitative way [26,27]. Furthermore, MA allows us to explore the heterogeneity sources among the diverse studies, which helps to obtain additional information about the factors contributing to the variability of the observed outcomes in response to a specific treatment [28]. MA has been frequently used in biomedical and clinical research, but its use in research related to secondary plant metabolites and meat science is still limited [29]. The objective of this meta-analysis was to evaluate the effect of dietary supplementation with tannins on productive performance, carcass characteristics, meat quality and oxidative stability, and antioxidant status of sheep blood plasma. The heterogeneity of responses was also examined using meta-regression analysis with the purpose of identifying factors contributing to the variability in the response variables.

## 2. Materials and Methods

### 2.1. Literature Search and Study Selection

To perform a robust meta-analysis, the Preferred Reporting Items for Systematic Reviews and Meta-Analyses (PRISMA) guidelines [30] were used in the identification, selection, choice and inclusion of information, as shown in Figure S1. To identify studies that evaluated the effect of supplementation with TANs on productive performance, carcass and meat quality characteristics, and antioxidant status of sheep blood serum, a systematic literature search was performed in the scientific databases of Scopus, ScienceDirect, Web of Science, and PubMed. The following keywords were used in all databases: tannin, lamb, sheep, growth performance, intake, carcass characteristics, meat quality, antioxidant status, and oxidative stability. The search and selection process was limited to the results of papers published between January 2010 and June 2021, where 1157 scientific publications were identified (Figure S1). These publications went through a two-step selection process as previously described by other authors [31,32]. First, a selection of titles and abstracts was performed excluding simulation studies, review articles, studies not conducted in sheep, in-vitro studies, and articles that did not include the variables of interest. Subsequently, to be considered, studies had to meet inclusion criteria previously used by other authors [31,32,33]: (1) Use of sheep and specify the procedure used to randomly assign animals within treatments; (2) data on productive performance, oxidative stability of meat, meat and/or carcass quality characteristics, or blood serum antioxidant status; (3) similarity between control and experimental groups except for the presence of TANs; (4) quantification or possible determination of the amount of TANs in the diet; (5) peer-reviewed journal articles written in English; (6) least squares means of the control and experimental groups with measures of variability (standard deviation or standard error); and (7) sample size.

### 2.2. Data Extraction

After exclusion of duplicate papers and selection of titles and abstracts, 99 full-text articles were evaluated; of these, only 53 articles met the inclusion criteria (Table A1) and were used to obtain the quantitative data for the meta-analysis. To be considered, variables had to be reported in at least three studies [32,34]. Consequently, the response variables included in the meta-analysis were: daily weight gain, dry matter intake, feed conversion rate (feed intake/weight gain), hot and cold carcass yield, backfat thickness, *Longissimus dorsi* muscle area, meat quality characteristics (pH, color, chemical composition, malondialdehyde content, among others), as well as total antioxidant capacity, malondialdehyde content (as an indicator of lipid oxidation) and antioxidant enzyme activity (superoxide dismutase, catalase and glutathione peroxidase) in blood serum. In addition, when available, additional data were collected, such as: characteristics of the published study (author and year of publication), chemical composition of the diet, amount of forage in the diet (g kg^−1^ DM), number of replicates, amount of TANs in the diet (g kg^−1^ DM), period of supplementation with TANs (days), type of TANs (HTs, CTs or mixture of both), source of botanical origin of the TANs, and method of inclusion of the TANs (extract or naturally present in the diet). The references of the articles included in the dataset are listed in Table A1. Averages, standard deviation (SD), and number of replicates for each treatment were extracted from these articles. When the articles presented the SD of each experimental group, these values were used directly in the meta-analysis. Where the SD was not reported, it was calculated by multiplying the standard error of the means (SEM) by the square root of the sample size, using the equation: SD = SEM × √n, as previously reported by Higgins and Thomas [35], where *n* = number of replicates.

### 2.3. Calculations and Statistical Analysis

Meta-analysis and meta-regression data were analyzed using the Open Meta-analyst for Ecology and Evolution software [36]. Response variables were analyzed using the standardized mean difference (SMD), also referred to as effect size (ES), in which the difference between the means of the experimental and control groups was standardized using the SD of the groups with and without TANs [37]. SMDs were calculated using methods previously described by DerSimonian and Laird [38] for random-effects models. The SMD is a more robust estimate of ES when heterogeneity exists in the data set [39]. The variables of the chemical composition of the diets were analyzed with the MEANS procedure using the SAS statistical program [40] to obtain descriptive statistics values. Differences in the composition of the diets of the control and TANs-supplemented treatments were evaluated by the MIXED procedure, using the studies as random effect and Tukey’s test to detect differences between treatments, as previously reported by other authors [32,41].

### 2.4. Heterogeneity

Heterogeneity was measured using the I^2^ statistic and the chi-square (Q) test [42]. Because of the relatively low capacity of the Q test to detect heterogeneity among a small number of treatment comparisons, an α level of 0.10 was used [39,43]. I^2^ (percentage of variation) values range from 0 to 100%, where values close to 25% indicate low heterogeneity, close to 50% indicate moderate heterogeneity, and close to 75% indicate high heterogeneity between studies [26,28]. Likewise, I^2^ values greater than 50% indicate significant heterogeneity [33].

### 2.5. Publication Bias

Since a visual inspection of funnel plots (generally used to assess publication bias) is subjective and must be balanced with additional analyses [44], three methods were used to assess evidence of publication bias: (1) the funnel plot [45]; (2) Begg’s adjusted rank correlation [46]; and (3) Egger’s regression asymmetry test [47]. Bias was considered to be present when the funnel plot showed asymmetry, or when at least one of the statistical methods (Begg’s test and Egger’s test) was significant (*p* < 0.10). Tests to assess publication bias are inappropriate when the variable to be assessed is not reported in at least 10 studies, and when significant heterogeneity (Q) is detected with an α ≤ 0.10, because it may lead to false-positive claims [48]. Consequently, funnel plots, Begg’s test, and Egger’s test were only performed for variables that met the aforementioned criteria. In cases where statistical evidence of publication bias was found, the “trim-and-fill” method of Duval and Tweedie was used to estimate the number of possible missing observations [49].

### 2.6. Meta-Regression

Sources of parameter heterogeneity that showed Q with an α level of ≤ 0.10 [43] or I^2^ greater than 50% [26] were assessed by meta-regression analysis. Similar to the publication bias tests, meta regression analysis was only performed for response variables that were reported in at least 10 studies [44]. The meta regression was estimated using the DerSimonian and Laird method of moments, which is well known for estimating variance among studies [26]. Continuous and categorical variables were used in the meta-regression. The continuous variables included: differences in neutral detergent fiber (NDF) and ether extract (EE) content in the diets (g kg^−1^ DM), TANs dose (g kg^−1^ DM), and duration of the experimental phase (days). Categorical variables included: source of botanical origin of TANs, method by which TANs were supplied (extract or as part of some dietary ingredient), type of TANs (CTs, HTs or mixture of both), and age of animals (≤3 months of age or >3 months of age). When categorical covariates were significant at an α level of ≤0.05, SMD was assessed by subgroup analysis [32,41]. Likewise, when the meta regression was significant (*p* ≤ 0.05) for supplementation level and experimental period, these covariates were evaluated by subgroup analysis by dividing the covariates as follows: dietary TANs supplementation level (≤20 or >20 g kg^−1^ DM); and experimental period (≤70 or >70 days).

## 3. Results

### 3.1. Study Attributes and Excluded Studies

Descriptive statistics and mean test for diet composition are presented in Table 1. Except for NDF, EE and organic matter (OM) content, no significant differences were observed between the control and TANs treatment for the rest of the nutritional components of the diet. Among the nutritional components, only fiber and fat content seem to have considerable effects on productive performance, carcass characteristics and meat quality [50]. Thus, it is possible to exclude the effects of the rest of the diet components on the response of animals to TANs supplementation for the data set.

The studies included in the present meta-analysis were conducted in 21 different countries, predominantly in Brazil (17%) and Iran (13.2%). The experimental doses of TANs ranged from 0.02 to 132 g kg^−1^ DM, and the duration of the experimental periods varied from 28 to 180 days (Table 1). The TANs used were divided into: CTs, HTs and mixture of both. Of the treatments, 54.7% used mixtures of CTs and HTs, 39.6% used CTs and the remaining 5.7% used HTs. Moreover, 79.3% of the treatments used plant parts, forages or by-products containing TANs in natural form, and 20.7% used extracts of TANs in the diets. The studies included in the meta-analysis used a total of 36 different sources of TANs, the majority of treatments (13.2%) used TANs from Vitis vinifera, 9.4% from Cistus ladanifer, 7.5% from plant mixtures and 9.4% from pomegranate (Punica granatum) and in the other 60.5% of the treatments, 32 other different sources of TANs were used.

### 3.2. Growth Performance and Carcass Characteristics

Table 2 shows that dietary supplementation with TANs increased (*p* < 0.05) daily weight gain (DWG), hot carcass yield (HCY), cold carcass yield (CCY), backfat thickness (BFT) and *Longissimus dorsi* muscle area (LMA). There was no observed a significant impact of the inclusion of TANs in the diet on dry matter intake (DMI; *p* > 0.05). In addition, feed conversion ratio (FCR) decreased in response to dietary supplementation with TANs (*p* < 0.05).

### 3.3. Meat Quality Characteristics

No significant effects of TANs inclusion in the sheep diet (*p* > 0.05) were observed on meat pH, meat lightness (L*) and yellowness (b*), Warner–Bratzler shear force (WBSF), meat cooking loss (CL), protein content, intramuscular fat (IMF) and meat ash (Table 3). Dietary supplementation with TANs decreased drip loss (DL) and meat moisture (*p* < 0.05). While meat redness (a*) increased in response to dietary supplementation with TANs (*p* < 0.05). In addition, malondialdehyde content in raw meat (MDAc) and metmyoglobin (MetMb) content of meat decreased in response to dietary supplementation with TANs (*p* < 0.05).

### 3.4. Antioxidant Status

Table 4 shows that dietary supplementation with TANs increased total antioxidant capacity (TAC), and catalase (CAT) and glutathione peroxidase (GPx) enzyme activity in blood serum (*p* < 0.05). On the other hand, the concentration of malondialdehyde in blood serum (MDAs) decreased (*p* < 0.05; Table 2) in response to TANs’ supplementation. Moreover, no significant impact was observed for blood serum superoxide dismutase (SOD) enzyme activity (*p* > 0.05).

### 3.5. Analysis of Publication Bias

DWG, DMI, FCR, HCY, LMA, meat pH, meat color (L*, a* and b*), WBSF, CL, IMF and MDAc had significant heterogeneity (Q) with an α ≤ 0.10. Whereas CCY, BFT, DL, moisture, protein, ash, MetMb, TAC, SOD, CAT, GPx and MDAs were reported in less than 10 studies. Therefore, tests to assess publication bias were not performed for any variable, because under these conditions they may result in false positive claims [48].

### 3.6. Meta-Regression

Significant heterogeneity (Q; *p* < 0.10) was observed for DWG, DMI, FCR, HCY, LMA (Table 2), meat pH, meat color (L*, a* and b*), WBSF, DL, CL, moisture content, protein, IMF, meat ash, MDAc, MetMb (Table 3), TAC, SOD, CAT, and MDAs (Table 4). Since it is not advisable to use meta-regression when there are fewer than 10 studies that reported the response variable of interest [44], this analysis was only performed for the variables: DWG, DMI, FCR, HCY, LMA, meat pH, L*, a*, b*, CL, IMF, and MDAc.

Table 5 shows that the dose of TANs explained (*p* < 0.05) 0.54, 3.02, 8.84, 14.48, 1.26, 9.56, and 17.0% of the heterogeneity observed for DWG, DMI, FCR, HCY, CL, IMF, and MDAc, respectively. The period of TANs supplementation had a significant relationship with DWG, DMI, FCR, LMA, a*, and MDAc (*p* < 0.05); however, it only explained between 1.15 and 26.30% of the observed heterogeneity in these variables. Animal age was significantly related to FCR, LMA, a*, b*, IMF and MDAc, explaining 6.57, 66.18, 28.53, 2.80, 14.55 and 56.60% of the observed heterogeneity, respectively (*p* < 0.05). The type of TANs explained between 11.65 and 54.54% of the observed heterogeneity for FCR, meat pH, L*, b* and MDAc (*p* < 0.01). The method of inclusion of TANs in the diet explained 1.17, 23.06 and 5.11 of the observed heterogeneity in DWG, meat pH and MDAc, respectively (*p* < 0.05). The botanical origin of TANs had a significant relationship with DMI, FCR, HCY, LMA, meat pH, L*, b*, CL, IMF and MDAc, explaining between 1.12 and 100% of the observed heterogeneity in these variables (*p* < 0.01). A significant relationship (*p* < 0.001) was observed between a* and MDAc with dietary ether extract content (EED), where the variation in EED explained 19.28 and 4.60% of the heterogeneity observed in a* and MDAc, respectively. A significant relationship (*p* < 0.05) was observed between HCY and a* with the neutral detergent fiber content of the diets (NDFD), where variation in NDFD explained 6.27 and 21.86% of the heterogeneity observed in HCY and a*, respectively.

### 3.7. Subgroup Analysis

Table S1 shows that DWG increased when doses of TANs lower than 20 g kg^−1^ DM were used (SDM = 0.485; *p* < 0.001), but doses higher than 20 g kg^−1^ DM did not affect DWG (SMD = −0.282; *p* > 0.05). Similarly, DMI increased when doses of TANs lower than 20 g kg^−1^ DM were used (SDM = 0.324; *p* < 0.05), but doses higher than 20 g kg^−1^ DM did not affect DMI (SMD = −0.416; *p* > 0.05). Additionally, animals from studies using doses lower than 20 g kg^−1^ DM had lower FCR (SMD = −0.423; *p* < 0.001), but no differences were observed with respect to FCR in animals from studies using doses higher than 20 g kg^−1^ DM (SMD = 0.640; *p* > 0.05). HCY was higher in animals supplemented with doses of TANs lower than 20 g kg^−1^ DM (SMD = 0.276; *p* < 0.05), whereas doses higher than 20 g kg^−1^ DM did not modify HCY (SMD = 0.152; *p* > 0.05). CL increased when TANs doses lower than 20 g kg^−1^ DM were used (SMD = 0.501; *p* < 0.05) but decreased with doses higher than 20 g kg^−1^ DM (SMD = −1.158; *p* < 0.05). Low doses of TANs (<20 g kg^−1^ DM) did not affect IMF content (SMD = 0.207; *p* > 0.05), but doses higher than 20 g kg^−1^ DM reduced IMF content (SMD = −0.691; *p* < 0.001). MDAc decreased regardless of the dose of TANs used (*p* < 0.01); however, the effect was greater when doses lower than 20 g kg^−1^ DM were used (SMD = −2.735) compared to doses higher than 20 g kg^−1^ DM (SMD = −1.058). 

Table S2 shows that DWG increased in response to dietary supplementation with TANs regardless of the supplementation period (*p* < 0.05). However, the effect was greater when TANs were offered for more than 70 days (SMD = 0.515) compared to periods up to 70 days (SMD = 0.256). On the other hand, DMI increased when dietary supplementation with TANs lasted more than 70 days (SMD = 0.590; *p* < 0.05) but was not affected when TANs were offered for up to 70 days (SMD = −0.142; *p* > 0.05). In contrast, FCR decreased when dietary supplementation with TANs lasted more than 70 days (SMD = −0.549; *p* < 0.05) but was not affected when TANs were offered for up to 70 days (SMD = −0.197; *p* > 0.05). Additionally, a* of meat increased when dietary supplementation with TANs lasted more than 70 days (SMD = 0.981; *p* < 0.001) but was not affected when TANs were offered for up to 70 days (SMD = −0.029; *p* > 0.05). Higher LMA was observed when supplementation periods longer than 70 days were used (SMD = 0.870; *p* < 0.01), but when the period lasted less than 70 days no significant effects were observed in LMA (SMD = 0.063; *p* > 0.05). MDAc decreased in response to dietary supplementation with TANs regardless of the supplementation period used (*p* < 0.001). However, the effect was greater when TANs were offered for more than 70 days (SMD = −2.706) compared to periods up to 70 days (SMD = −0.784).

Table S3 shows that supplementation with TANs reduced FCR in animals older than three months of age (SMD = −0.519; *p* < 0.01), but no effect was observed in lambs up to three months of age (SMD = 0.099; *p* > 0.05). In contrast, LMA increased in sheep older than three months of age (SMD = 1.108; *p* < 0.001); however, supplementation with TANs did not affect LMA when lambs were up to three months of age (SMD = −0.245; *p* > 0.05). On the other hand, a* of meat increased when TANs were offered to sheep older than three months of age (SMD = 0.844; *p* < 0.001); however, supplementation with TANs did not affect a* of meat when sheep were up to three months of age (SMD = −0.061; *p* > 0.05). In contrast, meat b* decreased when TANs were offered to sheep up to three months of age (SMD = −0.246; *p* < 0.05), but no significant effects were observed in sheep older than three months of age (SMD = 0.502; *p* > 0.05). Dietary supplementation with TANs decreased IMF content in animals up to three months of age (SMD = −0.498; *p* < 0.01); nevertheless, when TANs were offered to sheep older than three months of age IMF content was not affected (SMD = 0.553; *p* > 0.05). Dietary supplementation with TANs reduced MDAc in animals older than three months of age (SMD = −4.489; *p* < 0.001), but no effect was observed in lambs up to three months of age (SMD = −0.320; *p* > 0.05).

Table S4 shows that FCR decreased in response to dietary supplementation of CTs (SMD = −0.563; *p* < 0.05) and HTs (SMD = −2.000; *p* < 0.001), but there was no change in FCR in sheep supplemented with mixtures of CTs and HTs (SMD = −0.093; *p* > 0.05). Meat pH decreased in response to dietary supplementation with HTs (SMD = −1.556; *p* < 0.001). However, there was no significant change in meat pH (*p* > 0.05) when CTs (SMD = −0.111) and mixtures of CTs and HTs (SMD = 0.128) were used. L* of meat increased when HTs were used (SMD = 1.373; *p* < 0.05), but there was no change (*p* > 0.05) when CTs (SMD = −0.072) and mixtures of CTs and HTs (SMD = −0.114) were used. Similarly, b* of meat increased when HTs were used (SMD = 3.312; *p* < 0.05) but decreased when CTs were used (SMD = −0.500; *p* < 0.001). However, meat b* was not affected in sheep supplemented with mixtures of CTs and HTs (SMD = 0.123; *p* > 0.05). Dietary supplementation with mixtures of CTs and HTs decreased MDAc (SMD = −3.666; *p* < 0.001), but there was no change (*p* > 0.05) of MDAc in sheep supplemented with CTs (SMD = −0.313) and HTs (SMD = −0.106).

Table S5 shows that DWG increased when ingredients containing TANs were supplied naturally in the diets (SMD = 0.422; *p* < 0.002) but DWG was not affected when TANs extracts were used (SMD = −0.233; *p* > 0.05). Meat pH was not affected by the method of inclusion of TANs in the diet (*p* > 0.05). MDAc decreased when ingredients containing TANs were supplied naturally in the diets (SMD = −2.664; *p* < 0.001) but was not affected when TANs extracts were used (SMD = −0.159; *p* > 0.05).

**Figure 1:**
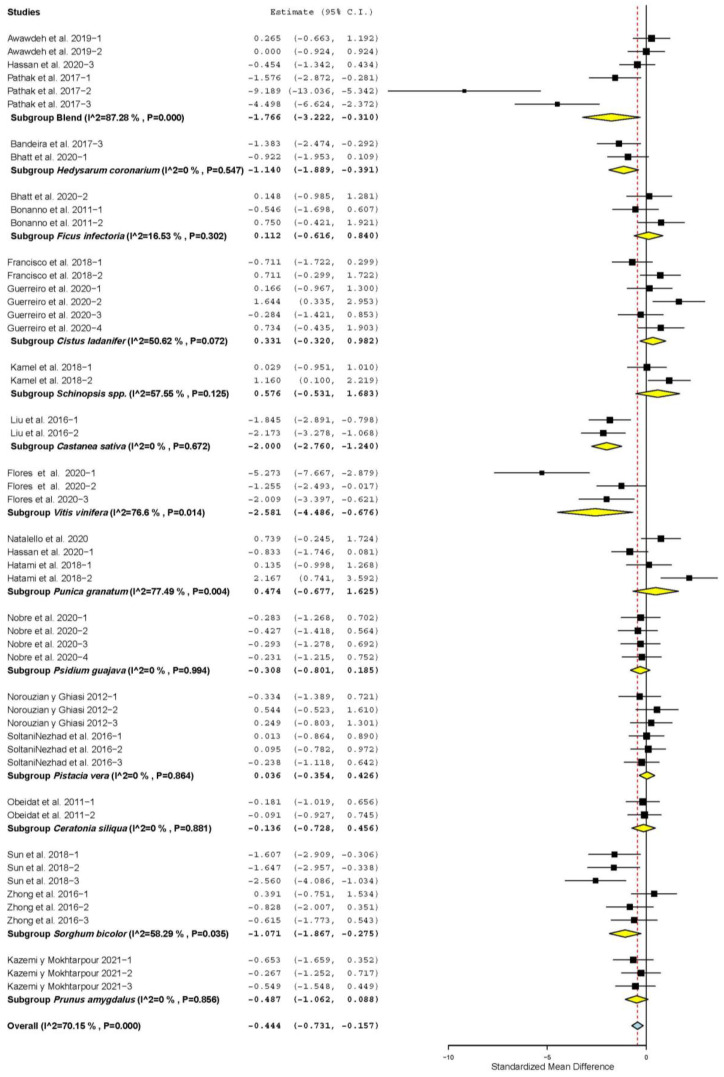
Forest plot of the effect size or standardized mean difference and 95% confidence interval of the botanical source of tannin on feed conversion ratio (FCR) in sheep. The solid vertical black line represents the mean difference of zero or no effect. Points to the left of the solid vertical black line represent reduction in FCR, while points to the right of the line indicate increase in FCR.

Figure S2 shows that DMI increased only when TANs came from *Mimosa tenuiflora* (SMD = 4.531; *p* = 0.001), *Hedysarum coronarium* (SMD = 7.809; *p* < 0.001), *Ficus infectoria* (SMD = 1.448; *p* < 0.001), *Schinopsis* spp. (SMD = 0.862; *p* = 0.026), *Pistacia vera* (SMD = 0.638; *p* = 0.038), *Passiflora edulis* (SMD = 0.872; *p* = 0.044) and *Prunus amygdalus* (SMD = 0.860; *p* = 0.036). However, it decreased when TANs came from *Cistus ladanifer* (SMD = −0.363; *p =* 0.050) and was not affected when TANs came from other plants (*p* > 0.05). Figure 1 shows that FCR decreased when TANs came from plant mixtures (SMD = −1.766; *p* = 0.017), *Hedysarum coronarium* (SMD = −1.140; *p* = 0.003), *Castanea sativa* (SMD = −2.000; *p* < 0.001); *Vitis vinifera* (SMD = −2.581; *p* = 0.008) and *Sorghum bicolor* (SMD = −1.071; *p* = 0.008). FCR was not affected in sheep supplemented with other sources of TANs (*p* > 0.05).

**Figure 2:**
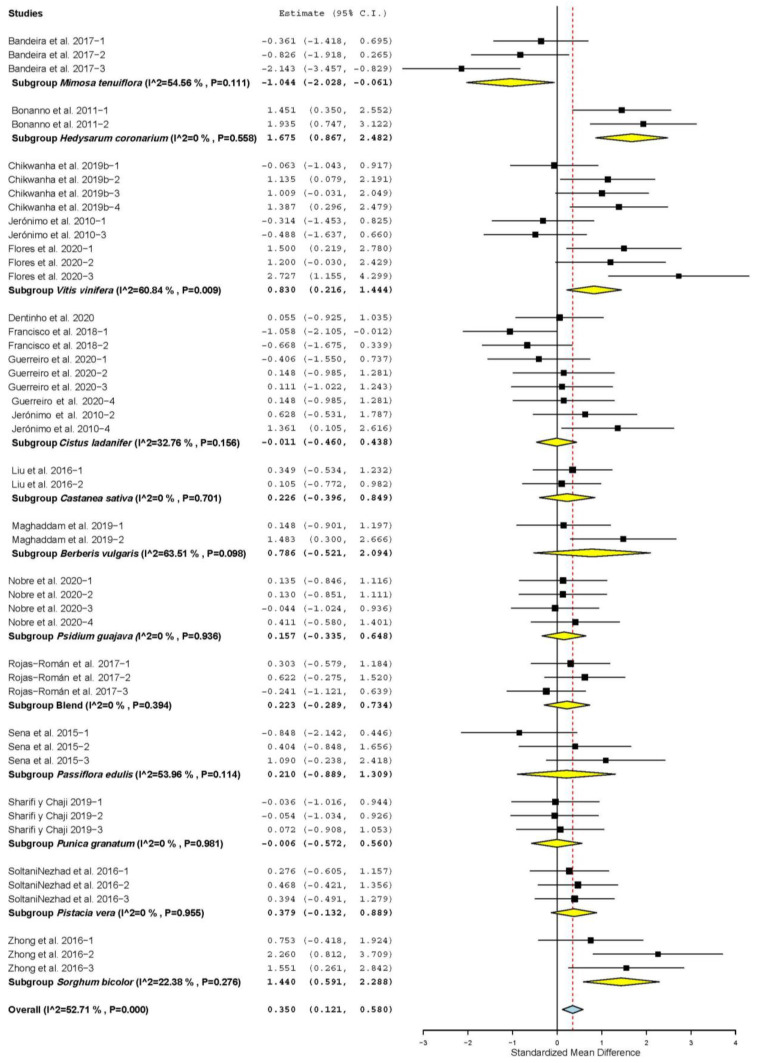
Forest plot of the effect size or standardized mean difference and 95% confidence interval of the botanical source of tannin on hot carcass yield (HCY) of sheep. The solid vertical black line represents the mean difference of zero or no effect. Points to the left of the solid vertical black line represent reduction in HCY, while points to the right of the line indicate increase in HCY.

Figure 2 shows that HCY only increased when TANs were from *Hedysarum coronarium* (SMD = 1.675; *p* < 0.001), *Vitis vinifera* (SMD = 0.830; *p* = 0.008) *and Sorghum bicolor* (SMD = 1.440; *p* < 0.001). However, HCY decreased when TANs were from *Mimosa tenuiflora* (SMD = −1.044; *p* = 0.037). HCY was not affected when other plants were used as sources of TANs (*p* > 0.05).

**Figure 3:**
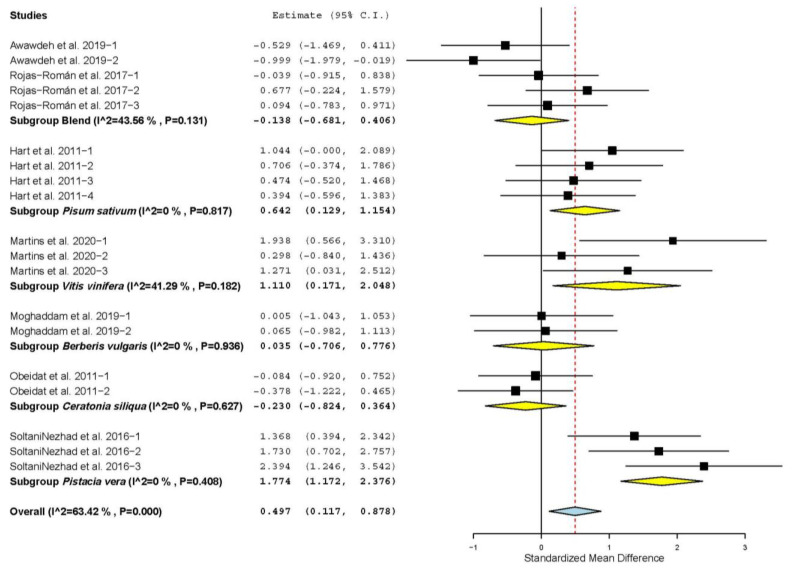
Forest plot of the effect size or standardized mean difference and 95% confidence interval of the botanical source of tannin on sheep *Longissimus dorsi* muscle area (LMA). The solid vertical black line represents the mean difference of zero or no effect. Points to the left of the solid vertical black line represent reduction in LMA, while points to the right of the line indicate increase in LMA.

Figure 3 shows that LMA increased only when *Pisum sativum* (SMD = 0.642; *p* = 0.014), *Vitis vinifera* (SMD = 1.110; *p* = 0.021) and *Pistacia vera* (SMD = 1.774; *p* < 0.001) were used as sources of TANs. However, LMA was not affected when other plants were used as sources of TANs (*p* > 0.05).

**Figure 4:**
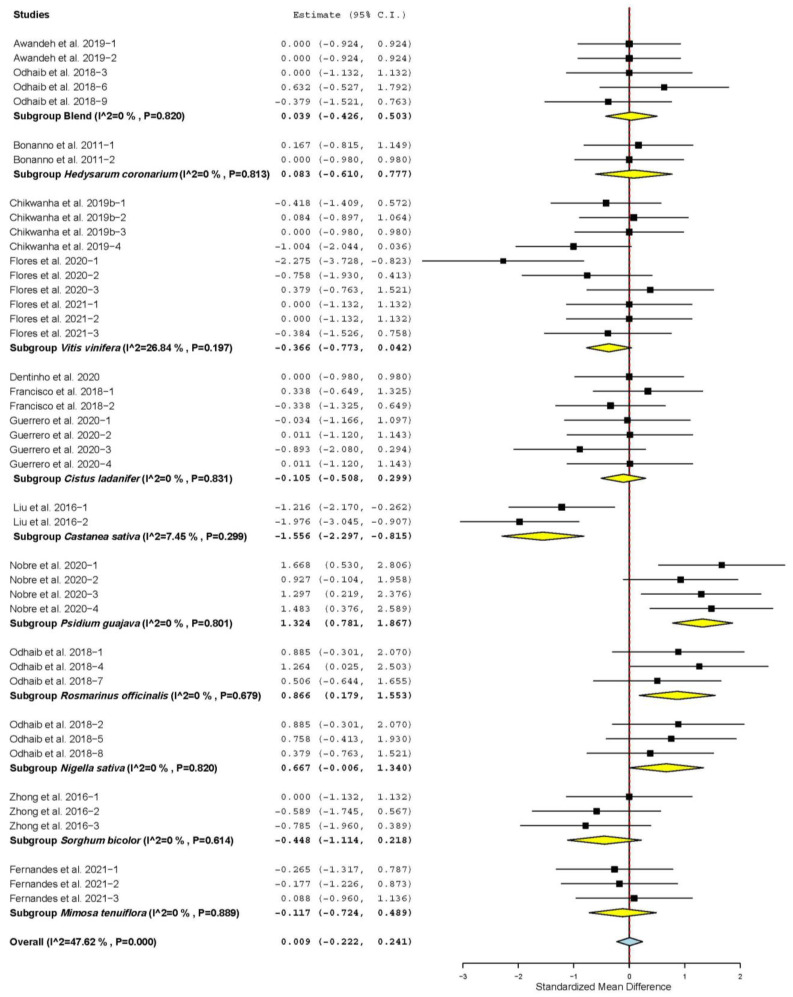
Forest plot of the effect size or standardized mean difference and 95% confidence interval of the botanical source of tannin on meat pH of sheep. The solid vertical black line represents the mean difference of zero or no effect. Points to the left of the solid vertical black line represent reduction in meat pH, while points to the right of the line indicate increase in meat pH.

Figure 4 shows that meat pH increased only when TANs came from *Psidium guajava* (SMD = 1.324; *p* < 0.001), *Rosmarinus officinalis* (SMD = 0.866; *p* = 0.013) and *Nigella sativa* (SMD = 0.667; *p* = 0.050). Meat pH decreased when TANs were from *Castanea sativa* (SMD = −1.556; *p* < 0.001), and it was not affected when TANs were from other sources (*p* > 0.05).

Figure S3 shows that L* of meat increased only when *Cistus ladanifer* (SMD = 0.470; *p* = 0.028), *Castanea sativa* (SMD = 1.803; *p* = 0.036) and *Mimosa tenuiflora* (SMD = 0.851; *p* = 0.009) were used as sources of TANs. L* decreased when TANs were from *Sorghum bicolor* (SMD = −0.947; *p =* 0.007), and it was not affected by other sources of TANs (*p* > 0.05). In contrast, Figure S4 shows that b* of meat decreased only when *Acacia mearnsii* (SMD = −0.884; *p* = 0.042), *Ceratonia siliqua* (SMD = −0.618; *p* = 0.045) and *Sorghum bicolor* (SMD = −1.358; *p* < 0.001) were used as sources of TANs. However, b* increased when TANs were from *Mimosa tenuiflora* (SMD = 1.903; *p* = 0.033), and it was not affected when TANs were from other sources (*p* > 0.05).

**Figure 5:**
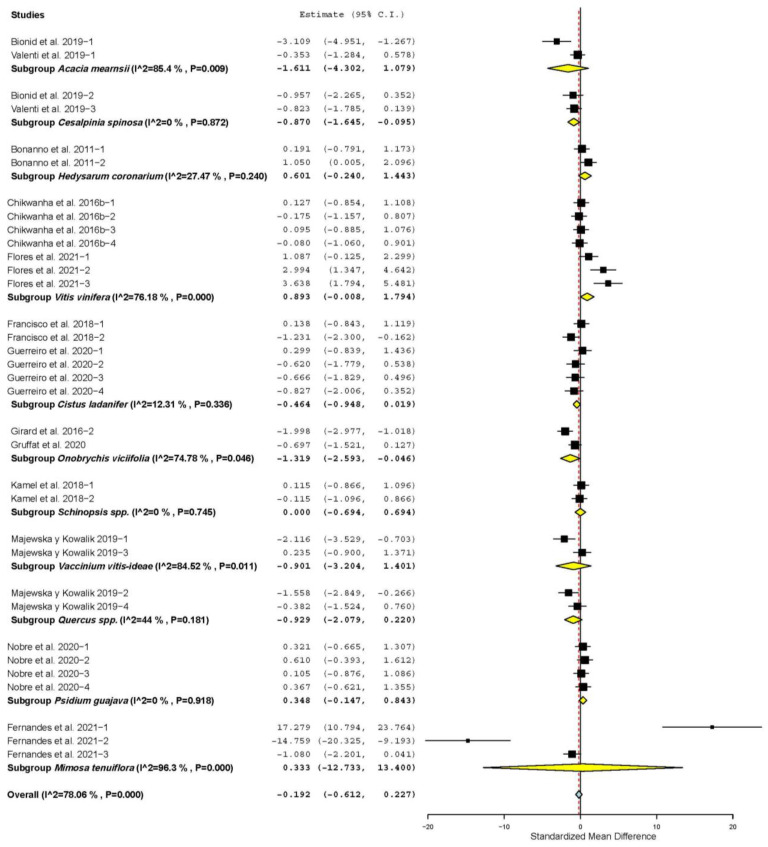
Forest plot of the effect size or standardized mean difference and 95% confidence interval of the source of botanical origin of tannin on intramuscular fat (IMF) content in sheep meat. The solid vertical black line represents the mean difference of zero or no effect. Points to the left of the solid vertical black line represent reduction in IMF, while points to the right of the line indicate increase in IMF.

Figure 5 shows that the IMF content of meat decreased only when *Cesalpinia spinosa* (SMD = −0.870; *p* = 0.028) and *Onobrychis viciifolia* (SMD = −1.319; *p* = 0.042) were used as sources of TANs. However, IMF increased when TANs were from *Vitis vinifera* (SMD = 0.893; *p* = 0.050), and it was not affected when TANs were from other plants (*p* > 0.05). Additionally, Figure S5 shows that CL decreased only when TANs came from *Vitis vinifera* (SMD = −1.584; *p* = 0.047) and *Rosmarinus officinalis* (SMD = −0.691; *p* = 0.045). CL increased when TANs came from *Castanea sativa* (SMD = 1.949; *p* < 0.001) and *Psidium guajava* (SMD = 6.842; *p* < 0.001), and it was not affected when TANs came from other sources (*p* > 0.05).

**Figure 6:**
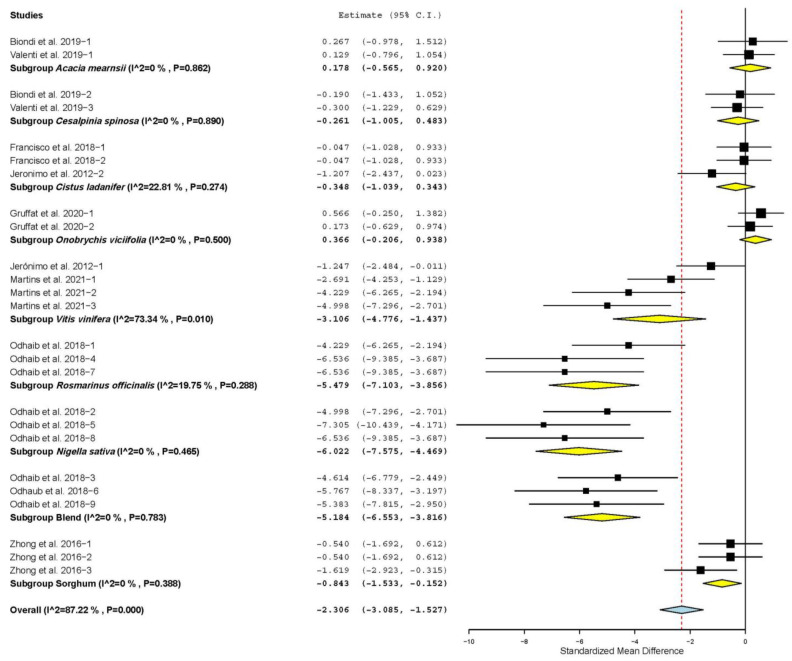
Forest plot of the effect size or standardized mean difference and 95% confidence interval of the source of botanical origin of tannin on the malondialdehyde content of raw sheep meat (MDAc). The solid vertical black line represents the mean difference of zero or no effect. Points to the left of the solid vertical black line represent reduction of MDAc, while points to the right of the line indicate increase of MDAc.

Figure 6 shows that MDAc content decreased only when TANs came from *Vitis vinifera* (SMD = −3.106; *p* < 0.001), *Rosmainus officinalis* (SMD = −5.479; *p* < 0.001), *Nigella sativa* (SMD = −6.022; *p* < 0.001), *Sorghum bicolor* (SMD = −0.843; *p* = 0.017) and plant mixtures (SMD = −5.184; *p* < 0.001). While MDAc was not affected when TANs were from other plants (*p* > 0.05).

## 4. Discussion

It has been suggested that high doses of TANs in the diet (>55 g kg^−1^ DM) may have negative effects on feed utilization efficiency and growth rate of ruminants; however, low-moderate levels (20 to 40 g kg^−1^ DM) could result in neutral or even positive effects [9,12]. In this regard, a meta-analysis conducted by Orzuna-Orzuna et al. [32] reported that dietary supplementation with TANs at average doses of 14.61 g kg^−1^ DM did not affect DWG or feed efficiency in beef cattle. However, in the present meta-analysis, higher DWG and lower FCR were observed in response to dietary supplementation with TANs. This suggests that TANs improve growth rate and feed utilization efficiency in sheep. The lower FCR observed could be explained because TANs supplementation reduces infection by gastrointestinal nematodes, which decrease feed efficiency in livestock [1]. According to Pimentel et al. [12], dietary inclusion of TANs in moderate doses can increase the efficiency of nutrient utilization of the diet, due to the ability of TANs to form complexes with macromolecules. In addition, the presence of TANs in diets consumed by ruminants has been reported to reduce enteric methane production [32], increase duodenal flux of amino acids and microbial protein [51], and reduce energy losses due to urea excretion [32,52]. This would partially explain the positive effects observed in DWG and FCR for sheep supplemented with TANs. On the other hand, reactive oxygen species (ROS) oxidize and destroy cellular biological molecules and impair the integrity of the intestinal membrane, resulting in reduced nutrient absorption [53,54]. Antioxidant enzymes and exogenous antioxidants can help restore oxidative balance and maintain healthy intestinal mucosa [21,54,55]. Consequently, the positive effects observed for DWG and FCR in the present study suggest that TANs reduced the presence of ROS and intestinal membrane damage in sheep. This hypothesis is supported by the observed increase in CAT and GPx in sheep supplemented with TANs, because CAT and GPx can convert ROS into less harmful compounds for the organism and prevent lesions in the gastrointestinal mucosa [53,56].

Although some review articles have suggested that the presence of TANs in the diet may negatively affect DMI in ruminants [1,9,57], in the present meta-analysis no changes in DMI were observed in response to dietary supplementation with TANs. This absence of changes in DMI probably occurred because the average dose of TANs used was 19.29 g kg^−1^ DM and negative effects of TANs on intake seem to occur with doses higher than 50 g kg^−1^ DM [4,9,12]. Similar to our results, two previously elaborated meta-analyses reported that dietary supplementation with TANs at average concentrations of 9.5 and 14.61 g kg^−1^ DM did not significantly affect DMI of dairy cows in production and beef cattle, respectively [31,32]. These results together suggest that TANs can be used in sheep and cattle without negative effects on feed intake.

It has been suggested that the reduction of feed palatability could be produced by a reaction between dietary TANs and salivary mucoproteins, or by a direct reaction of TANs with taste receptors, causing an astringent sensation [10,58,59]. However, prolonged exposure to dietary TANs can induce adaptive mechanisms in ruminants, such as changes in the amount of proline and other salivary proteins with high affinity for TANs [8,59,60,61]. Unlike other protein complexes and TANs formed, protein complexes rich in proline and TANs are stable over the entire pH range of the digestive tract, which could eliminate or reduce their negative effect on palatability and feed intake [10,62,63,64]. Regarding this, in the present study a subgroup analysis revealed that DMI was not affected when TANs were offered for up to 70 days but increased when supplementation lasted more than 70 days.

With respect to carcass characteristics, dietary supplementation with TANs increased HCY and CCY. There is limited information on the effects of TANs on ruminant carcass characteristics, which makes it difficult to explain the results observed in the present meta-analysis. However, variation in carcass fat and muscle deposition can modify carcass performance [65]. Consequently, the observed increase in LMA and BFT in the carcasses of sheep supplemented with TANs could partially explain the higher HCY and CCY obtained in the present meta-analysis.

BFT and LMA also increased in response to dietary supplementation with TANs. The mechanism of action of TANs on lipogenesis and muscle development has not been studied in sheep. Nevertheless, some polyphenolic compounds have been reported to increase BFT in beef cattle by changing the differential expression of genes involved in lipid metabolism [66]. Similarly, dietary supplementation from plants with phenolic compounds can increase muscle fiber size and increase skeletal muscle mass in lambs [67]. Similar effects of TANs consumption in the present meta-analysis would partially explain the observed increases in BFT and LMA.

Meat with high pH has higher microbial deterioration, which reduces its quality and shelf life [50]. Although dietary supplementation with TANs did not affect meat pH, subgroup analysis revealed that meat pH decreased significantly when HTs were used. This suggests that HTs could reduce microbial spoilage of meat, and consequently improve its quality and increase its shelf life. In this regard, Biondi et al. [68] observed lower overall load of pathogenic bacteria (*Escherichia coli*, *Enterobacteriaceae* and *Pseudomonas* spp.) in meat from lambs supplemented with HTs (40 g kg^−1^ DM) of *Caesalpinia spinosa* and concluded that HTs may have antimicrobial activity within muscle tissue.

Color is one of the most important characteristics that determines meat quality, because it is the first attribute that attracts consumers when choosing fresh meat [50,69]. The pH, IMF content, the amount of myoglobin, and the formation and accumulation of muscle metmyoglobin are the main factors that influence the color of small ruminant meat [65]. Particularly, L* of meat depends on the IMF content [70] and is negatively correlated (r = −0.63) with muscle myoglobin concentration [71]. Similarly, b* of meat is correlated with pH [72], and with the IMF content of meat [70]. In the present meta-analysis, pH and meat IMF content were similar between treatments. In addition, previous studies [17,18] have reported that dietary supplementation of TANs does not affect muscle myoglobin content in sheep. These findings could partially explain the absence of L* and b* changes observed in sheep consuming TANs in the present study. Additionally, compounds originating in meat as a consequence of lipid oxidation have been reported to promote the formation of metmyoglobin, which reduces a* values in meat [73]. In the present meta-analysis, MDAc (as an indicator of lipid oxidation in meat) and the metmyoglobin content of meat decreased in response to dietary supplementation with TANs, which would partially explain the observed increase in a* in meat from sheep supplemented with TANs.

Meat tenderness is one of the main characteristics that influences consumers’ meat choice and can be evaluated by WBSF [65]. It has been hypothesized that some natural antioxidants, such as polyphenols extracted from citrus fruits, might contain tenderizing compounds because their use can increase meat tenderness [74]. However, although TANs are polyphenolic compounds with antioxidant activity [75], in the present study the addition of TANs in the sheep diet did not affect WBSF. These results suggest that TANs do not affect the tenderness of sheep meat. Malheiros et al. [76] reported that ROS production can improve meat tenderness by increasing the degradation of toughness-related structural proteins. It has been reported that TANs can improve the antioxidant status of small ruminant meat by increasing mRNA and protein expression levels of SOD and GPx in skeletal muscle cells [77]. This effect could reduce structural and functional damage in muscle cells and tissues caused by ROS [78], which would partially explain the absence of changes observed for WBSF in the present study.

DL and CL are parameters used to evaluate the water holding capacity (WHC) of meat [65]. In the present meta-analysis, the values observed for DL suggest that dietary supplementation with TANs can improve WHC. However, these results should be interpreted carefully due to the low number of studies that reported on this variable. There is a strong negative correlation (r = −0.894) between WHC and CL [79]. Therefore, the results observed for CL suggest that TANs do not affect WHC of sheep meat. Meat pH is also related to WHC [50,65], and variation in IMF content can alter muscle structure and modify water retention in meat [65]. This suggests that the similarity of IMF content and meat pH between treatments could be related to the lack of changes observed for CL in the present meta-analysis.

Meat moisture content decreased in response to dietary supplementation with TANs, suggesting that TANs might affect WHC of meat. However, these results should be interpreted carefully due to the low number of studies that reported on this variable. Moreover, the results observed for protein, IMF and ash content of meat indicate that supplementation with TANs does not affect the nutritional composition of sheep meat.

Subgroup analysis revealed that doses of TANs higher than 20 g kg^−1^ DM can reduce IMF content. The mechanism of action of TANs on IMF deposition has not been studied in sheep. However, the number and size of intramuscular adipocytes have been reported to be related to the process of IMF deposition or reduction in livestock [80,81]. Gallic acid (a typical isomer of HTs) can inhibit bovine adipocyte proliferation and adipogenesis under in-vitro conditions [81]. Similarly, in-vitro studies have reported that CTs and HTs inhibit preadipocyte differentiation [82,83], and CTs induce apoptosis in mature adipocytes and inhibit lipid accumulation in maturing preadipocytes [82]. This would partly explain the lower IMF content in meat from sheep supplemented with more than 20 g kg^−1^ DM of TANs. The lower IMF content could be related to the reduced moisture content of meat from sheep supplemented with TANs, because there is a negative correlation (r = −0.47) between moisture content and IMF content of meat [84].

Oxidation reactions during the processing, distribution and storage of meat products can cause physicochemical changes and undesirable odors that affect the quality of the final product [85]. For example, oxidation of myoglobin and lipids can cause discoloration and off-flavor development in meat, respectively [86]. In the present meta-analysis, lipid oxidation and myoglobin oxidation of meat decreased in response to dietary supplementation with TANs. This suggests that TANs may induce antioxidant enzyme gene expression in sheep muscle [87], similar to what was previously observed by Zhong et al. [77] in skeletal muscle cells from goat treated with *Camellia sinensis* TANs under in-vitro conditions. These results also suggest that TANs could be used to delay the discoloration and appearance of off-flavor in meat, and consequently improve the quality and shelf life of meat products.

TANs are polyphenolic compounds with antioxidant activity [75]. It has been reported that phenols can switch from an antioxidant to a prooxidant state when used in high doses [88]. In the present meta-analysis, lipid oxidation of meat decreased in response to dietary supplementation with TANs regardless of the dose used. However, the reduction was greater at doses below 20 g kg^−1^ DM. These results suggest that TANs may improve the oxidative stability of meat when supplied to the diet at low doses, but at high concentrations they could have prooxidant effects on meat, as previously reported for some essential oils rich in phenolic compounds [88].

Subgroup analysis revealed that lipid oxidation of meat decreased significantly only when TANs were fed to animals older than 3 months of age. This probably occurred because the presence of microorganisms in the rumen increases with the age of the animals [89], and the action of ruminal microorganisms increases the bioavailability of ingested TANs in sheep [90].

The type and source of TANs explained most (between 30 and 90%) of the heterogeneity observed in MDAc. These results suggest that the effects of TANs on oxidative stability of meat depend on the type and source of TANs used rather than the dose, period and method of supplementation. Subgroup analyses revealed that MDAc was significantly reduced only when mixtures of CTs and HTs were used. These results suggest a synergistic effect between both types of TANs in reducing lipid oxidation of meat. Although TANs have been shown to have high antioxidant activity [75], their effect on the antioxidant capacity of muscle tissues seems to depend on how effectively they can be absorbed through the gastrointestinal tract [91]. In this regard, it has been reported that some CTs can neither be degraded nor absorbed in the gastrointestinal tract of sheep [91]. In contrast, some HTs act for a short time because they are rapidly transferred to the blood plasma in sheep [90]. Similar effects of individual use of CTs and HTs in sheep would partially explain the results observed in the present meta-analysis.

TAC is an integrated parameter that considers all antioxidants present in blood plasma [92]. In the present study, TAC was observed to increase in response to dietary supplementation with TANs, suggesting that TANs intake may improve the total antioxidant status of sheep. Although there are no reference values for TAC in ruminants [93], TAC changes in blood plasma following supplementation with antioxidant-rich foods or purified antioxidants provide information on the absorption and bioavailability of ingested antioxidant compounds [92]. Therefore, although the metabolic fate of TANs ingested by ruminants is not yet fully understood [22], the results observed in the present meta-analysis suggest that TANs ingested by sheep may be degraded and absorbed in the gastrointestinal tract and subsequently transferred to the bloodstream to serve as exogenous antioxidants.

Excessive accumulation of prooxidant substances, such as ROS, can cause oxidative stress in ruminants [93,94]. Some antioxidant enzymes, such as GPx, SOD and CAT are important because they can convert ROS into less harmful compounds for the organism [56], and consequently can reduce ROS-mediated damage on biological macromolecules [95,96]. On the other hand, although ROS can attack any of the major biomolecules, lipids are particularly susceptible, so biomarkers of lipid peroxidation (e.g., malondialdehyde) are considered the best indicators of oxidative stress [97]. In the present meta-analysis, dietary supplementation with TANs increased the activity of antioxidant enzymes (CAT and GPx) and reduced the concentration of malondialdehyde in sheep blood serum. This suggests that inclusion of TANs in the diet could be used as a dietary strategy to mitigate oxidative stress in sheep, which could improve animal health [94].

## 5. Conclusions

The results of the present meta-analysis indicate that dietary supplementation with TANs does not affect dry matter intake, but improves daily weight gain, feed conversion ratio, total antioxidant capacity and antioxidant enzyme activity in sheep blood serum. The best result for daily weight gain is achieved using doses of TANs lower than 20 g kg^−1^ DM, with supplementation periods longer than 70 days and when TANs are supplied naturally as ingredients in the diet. The best results for feed conversion ratio are achieved using doses of TANs lower than 20 g kg^−1^ DM, with supplementation periods longer than 70 days, in animals older than three months of age and using CTs.

In addition, TANs reduce lipid oxidation in blood plasma and meat. The best results of lipid oxidation of meat are observed using mixtures of CTs and HTs, with supplementation periods longer than 70 days, using doses lower than 20 g kg^−1^ DM, in animals older than three months of age, and when TANs are supplied naturally as ingredients in the diet. Supplementation with TANs does not affect meat tenderness, chemical composition, pH and color (L* and b*). However, it increases hot and cold carcass yield, backfat thickness and *Longissimus dorsi* muscle area. The best hot carcass yield is obtained with *Vitis vinifera*, *Hedysarium coronarium* and *Sorghum bicolor* as sources of TANs. In contrast, the best *Longissimus dorsi* muscle area result is observed in animals older than three months of age, with supplementation periods longer than 70 days, and using *Vitis vinifera*, *Pisum sativum* and *Pistacia vera* as sources of TANs. In addition, supplementation with TANs improves meat a*, particularly with supplementation periods longer than 70 days and in animals older than three months of age.

## Figures and Tables

**Table 1:** Descriptive statistics of the complete data set for the effect of tannins supplementation to sheep diets.

Parameter		Mean		Median		Minimum		Maximum		SD	
Dietary Features	NC	Control	Tannin	Control	Tannin	Control	Tannin	Control	Tannin	Control	Tannin
Forage, g kg^−1^ DM	122	428.5	432.2	400.0	400.0	0	0	1000	1000	265.6	261.3
DM, g kg^−1^	100	874.8	871.7	900.0	903.4	160.0	156.0	953.9	947.5	122.8	130.8
OM, g kg^−1^ DM	46	857.5 ^b^	866.8 ^a^	910.0	922.0	148.0	146.0	957.2	984.5	193.7	188.7
CP, g kg^−1^ DM	124	156.3	156.9	157.0	156.5	84.0	89.0	251.0	255.0	26.9	26.5
EE, g kg^−1^ DM	106	35.5 ^b^	40.7 ^a^	29.3	36.3	13.0	12.8	81.0	98.3	16.6	18.3
NDF, g kg^−1^ DM	122	388.8 ^a^	375.9 ^b^	385.7	365.5	156.0	152.0	731.1	704.9	179.6	108.7
ADF, g kg^−1^ DM	94	211.2	212.1	190.0	179.5	81.0	69.8	516.0	496.5	94.1	94.2
Ca, g kg^−1^ DM	24	7.0	7.2	6.1	6.7	3.4	3.6	17.0	18.0	3.1	3.4
P, g kg^−1^ DM	22	4.0	4.0	4.4	4.4	2.0	2.0	5.8	6.2	1.2	1.3
ME, MJ kg^−1^ DM	56	10.6	10.6	10.5	10.7	9.2	8.8	12.5	12.6	0.8	0.9
Tannin, g kg^−1^ DM	135	-	20.2	-	15.5	-	0.02	-	132.0	-	20.6
Duration, days	135	70.0		70.0		28.0		180.0		30.0	

NC: number of comparisons; SD: standard deviation; DM: dry matter; OM: organic matter; CP: crude protein; EE: ether extract; NDF: neutral detergent fiber; ADF: acid detergent fiber; Ca: calcium; P: phosphorus; MJ: megajoule; ^a^,^b^: in the same row, means followed by different letters differ significantly by the Tukey test (*p* < 0.05).

**Table 2:** Growth performance and carcass characteristics of sheep supplemented with tannins.

					95% CI			Heterogeneity		
Parameter	N	NC	SMD	SE	Lower	Upper	*p*-Value	Q	*p*-Value	I^2^ (%)
Daily weight gain (DWG)	42	104	0.274	0.116	0.046	0.501	0.018	472.57	<0.001	78.20
Dry matter intake (DMI)	42	104	0.090	0.124	−0.152	0.333	0.466	524.508	<0.001	80.36
Feed conversion ratio (FCR)	27	60	−0.308	0.127	−0.556	−0.060	0.015	197.05	<0.001	70.06
Hot carcass yield (HCY)	26	59	0.234	0.108	0.023	0.445	0.030	142.03	<0.001	59.16
Cold carcass yield (CCY)	9	23	0.510	0.228	0.063	0.957	0.025	86.09	<0.001	74.45
Backfat thickness (BFT)	9	24	0.565	0.193	0.188	0.943	0.003	77.94	<0.001	70.49
*Longissimus dorsi* muscle area (LMA)	10	22	0.413	0.170	0.080	0.747	0.015	52.40	<0.001	59.92

N: number of studies; NC: number of comparisons; SMD: standardized mean difference; CI: confidence interval of SMD; SE: standard error. Q: chi-squared statistic and associated significance level (*p*-value); I^2^: percentage of variation.

**Table 3:** Meat characteristics of sheep supplemented with tannins.

					95% CI			Heterogeneity		
Parameter	N	NC	SMD	SE	Lower	Upper	*p*-Value	Q	*p*-Value	I^2^ (%)
Meat pH	19	52	0.037	0.098	−0.156	0.230	0.706	89.29	<0.001	42.88
Lightness (L*)	20	54	0.008	0.128	−0.243	0.260	0.950	151.88	<0.001	65.10
Redness (a*)	20	54	0.365	0.120	0.129	0.601	0.002	133.39	<0.001	62.27
Yellowness (b*)	20	54	0.048	0.145	−0.236	0.332	0.742	186.70	<0.001	71.61
WBSF	15	42	−0.027	0.093	−0.210	0.155	0.769	53.74	0.088	23.71
Drip loss (DL)	4	17	−2.828	0.516	−3.839	−1.817	<0.001	149.57	<0.001	89.30
Cooking loss (CL)	14	42	0.105	0.216	−0.317	0.528	0.626	243.00	<0.001	83.13
Moisture	5	16	−0.693	0.333	−1.345	−0.041	0.037	77.25	< 0.001	80.58
Protein	8	23	0.249	0.282	−0.304	0.802	0.378	114.45	<0.001	80.78
Intramuscular fat (IMF)	16	40	−0.168	0.186	−0.532	0.196	0.366	172.04	<0.001	77.33
Ash	6	20	0.507	0.332	−0.144	1.158	0.127	108.41	<0.001	82.47
Malondialdehyde (MDAc)	10	29	−2.020	0.326	−2.659	−1.380	<0.001	195.96	<0.001	85.65
Metmyoglobin (MetMb)	3	6	−0.482	0.222	−0.916	−0.047	0.030	5.25	0.387	4.68

N: number of studies; NC: number of comparisons; SMD: standardized mean difference; CI: confidence interval of SMD; SE: standard error. Q: chi-squared statistic and associated significance level (*p*-value); I^2^: percentage of variation; WBSF: Warner–Bratzler shear force.

**Table 4:** Oxidative status of lambs supplemented with tannins.

					95% CI			Heterogeneity		
Parameter	N	NC	SMD	SE	Lower	Upper	*p*-Value	Q	*p*-Value	I^2^ (%)
Total antioxidant capacity (TAC)	9	17	1.120	0.222	0.686	1.555	<0.001	43.661	<0.001	63.35
Superoxide dismutase (SOD)	6	14	−0.122	0.328	−0.766	0.521	0.709	61.306	<0.001	78.79
Catalase (CAT)	5	12	0.848	0.239	0.380	1.315	<0.001	22.963	0.018	52.10
Glutathione peroxidase (GPx)	3	6	0.801	0.209	0.392	1.211	<0.001	2.267	0.811	0
Malondialdehyde (MDAs)	7	17	−0.535	0.244	−1.014	-0.056	0.029	54.824	<0.001	70.81

N: number of studies; NC: number of comparisons; SMD: standardized mean difference; CI: confidence interval of SMD; SE: standard error. Q: chi-squared statistic and associated significance level (*p*-value); I^2^: percentage of variation.

**Table 5:** Meta-regression of the effects of dietary tannins supplementation on growth performance, meat quality and antioxidant status of sheep.

Parameter		Tannins Dose	Supplementation Period	Lamb’s Age	Tannins Type	Tannins Source	Method of Inclusion	EED	NDFD
DWG	QM	6.943	8.263	0.378	1.070	43.329	5.786	1.092	0.240
	df	1	1	1	2	32	1	1	1
	*p*-Value	0.008	0.004	0.989	0.586	0.087	0.016	0.296	0.624
	R^2^ (%)	0.54	2.85	0	0	0	1.17	0.92	0
DMI	QM	4.800	10.206	0.927	2.503	113.649	0.892	0.033	2.752
	df	1	1	1	2	32	1	1	1
	*p*-Value	0.028	0.001	0.336	0.286	<0.001	0.345	0.856	0.097
	R^2^ (%)	3.02	2.49	0	0	14.37	0	0	0
FCR	QM	7.711	3.716	5.348	9.193	48.362	0.129	0.006	0.335
	df	1	1	1	2	22	1	1	1
	*p*-Value	0.005	0.050	0.021	0.010	<0.001	0.720	0.940	0.563
	R^2^ (%)	8.84	3.35	6.57	13.11	29.30	0	0	0
HCY	QM	7.401	2.618	1.168	0.017	65.118	0.226	3.348	4.094
	df	1	1	1	2	22	1	1	1
	*p*-Value	0.007	0.106	0.280	0.992	<0.001	0.634	0.067	0.043
	R^2^ (%)	14.48	4.97	1.39	0	68.77	0	7.78	6.27
LMA	QM	0.004	5.968	13.647	0.100	36.514	0.625	2.658	2.251
	df	1	1	1	1	6	1	1	1
	*p*-Value	0.947	0.015	<0.001	0.751	<0.001	0.429	0.103	0.134
	R^2^ (%)	0	26.30	66.18	0	97.92	0	8.86	8.61
Meat pH	QM	0.453	1.354	0.076	17.572	68.102	6.236	0.852	0.016
	df	1	1	1	2	18	1	1	1
	*p*-Value	0.501	0.245	0.783	<0.001	<0.001	0.013	0.356	0.899
	R^2^ (%)	0	2.62	0	54.54	100	23.06	0	0
L*	QM	0.132	1.913	0.728	9.171	38.080	2.731	3.146	0.126
	df	1	1	1	2	19	1	1	1
	*p*-Value	0.716	0.167	0.393	0.010	0.006	0.098	0.076	0.723
	R^2^ (%)	0	0	0	11.65	26.61	1.34	0	0
a*	QM	0.003	13.834	16.603	4.698	29.143	0.435	12.199	10.968
	df	1	1	1	2	19	1	1	1
	*p*-Value	0.956	<0.001	<0.001	0.095	0.064	0.509	<0.001	<0.001
	R^2^ (%)	0	18.51	28.53	2.86	13.73	0	19.28	21.86
b*	QM	1.982	0.257	5.999	19.021	37.939	1.091	0.590	0.014
	df	1	1	1	2	19	1	1	1
	*p*-Value	0.159	0.612	0.014	<0.001	0.009	0.296	0.442	0.906
	R^2^ (%)	0	0	2.80	12.91	1.12	0	0	0
CL	QM	4.339	0.121	0.471	4.947	52.306	2.199	0.121	0.036
	df	1	1	1	2	14	1	1	1
	*p*-Value	0.037	0.728	0.492	0.084	<0.001	0.138	0.728	0.849
	R^2^ (%)	1.26	0	0	8.34	16.48	3.11	0	0
IMF	QM	3.967	0.419	7.676	0.866	80.997	2.191	1.462	3.435
	df	1	1	1	2	18	1	1	1
	*p*-Value	0.047	0.517	0.006	0.649	<0.001	0.139	0.227	0.064
	R^2^ (%)	9.56	0.38	14.55	0	54.24	0.33	1.96	2.56
MDAc	QM	9.33	11.05	56.60	29.38	143.390	9.83	13.23	0.31
	df	1	1	1	2	12	1	1	1
	*p*-Value	0.002	<0.001	<0.001	<0.001	<0.001	0.002	<0.001	0.574
	R^2^ (%)	17.00	1.15	56.60	32.50	93.81	5.11	4.60	4.73

QM: coefficient of moderators; QM was considered significant at *p* < 0.05; R^2^: amount of heterogeneity accounted for; df: degree of freedom; DWG: daily weight gain; DMI: dry matter intake; FCR: feed conversion ratio; HCY: hot carcass yield; LMA: *Longissimus dorsi* muscle area; L*: lightness; a*: redness: b*: yellowness; CL: cooking loss; IMF: intramuscular fat content; MDAc: malondialdehyde content in raw meat; EED: variation in ether extract content of the diets; NDFD: variation in neutral detergent fiber content of the diets.

## Data Availability

The datasets used and analyzed during the current study are available from the corresponding author on reasonable request.

## Supplementary Materials

The following are available online at https://www.mdpi.com/article/10.3390/ani11113184/s1, Figure S1: A PRISMA flow diagram detailing the literature search strategy and study selection for the meta-analysis. Table S1: Subgroup analysis of the effect of dietary tannin dose in sheep. Table S2: Subgroup analysis of the effect of tannin supplementation period in sheep. Table S3: Subgroup analysis of the effect of age on the response to tannin supplementation in sheep. Table S4: Subgroup analysis of the effect of tannin type in sheep. Table S5: Subgroup analysis of the effect of tannin inclusion method in sheep. Figure S2: Forest plot of the effect size or standardized mean difference and 95% confidence interval of the source of botanical origin of tannin on dry matter intake (DMI) in sheep. Figure S3: Forest plot of the effect size or standardized mean difference and 95% confidence interval of the botanical source of tannin on the lightness (L*) of sheep meat. Figure S4: Forest plot of the effect size or standardized mean difference and 95% confidence interval of the botanical source of tannin on the yellowness (b*) of sheep meat. Figure S5: Forest plot of the effect size or standardized mean difference and 95% confidence interval of the botanical source of tannin on cooking loss (CL) of sheep meat.

## Appendix A

**Table A1:** Summary of the studies included in the meta-analysis.

Author	Country	Tannin Source	Tannin Type	Method of Inclusion
Awawdeh et al. [98]	Jordan	B, B	B, B	N, N
Bandeira et al. [99]	Brazil	*Mimosa tenuiflora* (*n* = 3)	CT, CT, CT	N, N, N
Bhatt et al. [100]	India	PA, ER	B, B	N, N
Biondi et al. [68]	Spain	AM, CS	B, B	E, E
Bonanno et al. [101]	Italy	*Hedysarum coronarium*	CT, CT	N, N
Buccioni et al. [102]	Italy	CH, QU	HT, CT	E, E
Chikwanha et al. [103]	South Africa	VV (*n* = 4)	B, B, B, B, B	N, N, N, N, N
Chikwanha et al. [104]	South Africa	VV (*n* = 4)	B, B, B, B, B	N, N, N, N, N
Costa et al. [105]	Brazil	AM (*n* = 4)	CT, CT, CT, CT, CT	E, E, E, E, E
Dentinho et al. [20]	Brazil	CL	CT	E
Dey et al. [106]	India	*Ficus infectoria (n* = 3)	B, B, B	N, N, N
Abdalla et al. [107]	Brazil	OP, Clep	B, B	N, N
Fernandes et al. [14]	Brazil	*Mimosa tenuiflora* (*n* = 3)	B, B, B	N, N, N
Francisco et al. [108]	Portugal	CL (*n* = 2)	CT, CT	N, N
Girard et al. [109]	Switzerland	LC and OV	B, B	N, N
Guerreiro et al. [110]	Portugal	CL (*n* = 4)	CT, CT, CT, CT, CT	N, N, N, N, N
Gruffat et al. [111]	France	OV (*n* = 2)	CT, CT	N, N
Hart et al. [112]	United Kingdom	*Pisum sativum* (*n* = 4)	CT, CT, CT, CT, CT	N, N, N, N, N
Hassan et al. [113]	Egypt	*Punica granatum*, MI, B	B, B, B	N, N, N
Hatami et al. [114]	Iran	*Punica granatum* (*n* = 3)	B, B	N, N
Jerónimo et al. [115]	Portugal	VV (*n* = 2), CL (*n* = 2)	CT, CT, CT, CT, CT	E, N, E, N
Jerónimo et al. [116]	Portugal	VV (*n* = 2), CL (*n* = 2)	CT, CT, CT, CT, CT	E, N, E, N
Kamel et al. [117]	Saudi Arabia	QU (*n* = 2)	CT, CT	E, E
Kazemi and Mokhtarpour [118]	Iran	*Prunus amygdalus* (*n* = 3)	B, B, B	N, N, N
Leparmarai et al. [119]	Switzerland	VV	B	E
Lima et al. [120]	Brazil	*Macrotyloma axillare*	B	N
Liu et al. [21]	China	CH (*n* = 2)	HT, HT	E, E
López-Andrés et al. [91]	Italy	QU	CT	E
Majewska and Kowalik [121]	Poland	VAC, *Quercus* sp.	B, B	N, N
Flores et al. [122]	Brazil	VV (*n* = 3)	B, B, B	N, N, N
Flores et al. [123]	Brazil	VV (*n* = 3)	B, B, B	N, N, N
Moghaddam et al. [124]	Iran	*Berberis vulgaris* (*n* = 2)	B, B	N, N
Natalello et al. [18]	Italy	*Punica granatum*	B	N
Nobre et al. [125]	Brazil	*Psidium guajava* (*n* = 4)	B, B, B, B, B	N, N, N, N, N
Norouzian and Ghiasi [126]	Iran	*Pistacia vera (n* = 3)	B, B, B	N, N, N
Obeidat et al. [127]	Jordan	*Ceratonia siliqua (n* = 2)	CT, CT	N, N
Odhaib et al. [128]	Malaysia	RO (*n* = 3), NS (*n* = 3), B (*n* = 3)	B (*n* = 9)	N (*n* = 9)
Pathak et al. [13]	India	B, B	CT, CT	N, N
Peng et al. [95]	Canada	*Dalea purpurea*	CT	N
Po et al. [129]	Australia	*Ilex paraguarensis*	CT	N
Priolo et al. [16]	Italy	*Corylus avellana*	B	N
Rajabi et al. [130]	Iran	*Punica granatum* (*n* = 3)	B, B, B	N, N, N
Rojas-Román et al. [15]	Mexico	B, B, B	B, B, B	E, E, E
Sanchez et al. [131]	Mexico	*Caesalpinia coriaria*	B	N
Sena et al. [132]	Brazil	*Passiflora edulis* (*n* = 3)	CT, CT, CT	N, N, N
Sharifi and Chaji [133]	Iran	*Punica granatum* (*n* = 3)	B, B, B	N, N, N
SoltaniNezhad et al. [134]	Iran	*Pistacia vera (n* = 3)	B, B, B	N, N, N
Sun et al. [135]	China	*Sorghum bicolor* (*n* = 3)	CT, CT, CT	N, N, N
Valenti et al. [17]	Italy	AM, QU, CS	CT, HT, HT	E, E, E
Wang et al. [22]	Ireland	*Corylus avellana (n* = 2)	B, B	N, N
Yisehak et al. [136]	Ethiopia	*Albizia gummifera*	CT	N
Zhao et al. [137]	China	No reported	B, B	E, E
Zhong et al. [19]	China	*Sorghum bicolor* (*n* = 3)	CT, CT, CT	N, N, N

B: blend; PA: *Pimpinela anisum*; ER: *Eucalyptus rudis*; AM: *Acacia mearnsii*; CS: *Cesalpinia spinosa*; CH: chestnut (Castanea *sativa*); QU: quebracho (*Schinopsis* spp.); VV: *Vitis vinifera*; CL: *Cistus ladanifer*; OP: *Orbignya phalerata*; Clep: *Combretum leprosum*; LC: *Lotus corniculatus*; OV: *Onobrychis viciifolia*; MI: *Mangifera indica*; VAC: *Vaccinium vitis-ideae*; sorghum (*Sorghum bicolor*); HT: hidrolisable tannin; CT: condensed tannin; E: extract; N: naturally present. *n*: number of comparisons.
