# Supplementary material for: Growth Performance, Meat Quality and Antioxidant Status of Sheep Supplemented with Tannins: A Meta-Analysis
Source: Animals (Basel). 2021 Nov 8;11(11):3184. doi: 10.3390/ani11113184 (PMC8614576; doi:10.3390/ani11113184)
Supplement: Supplementary file 1 [file animals-11-03184-s001.zip › animals-1429497-supplementary.pdf]

animals

MDPI

Supplementary Materials

Growth Performance, Meat Quality and Antioxidant Status of Sheep Supplemented with Tannins: A Meta-Analysis

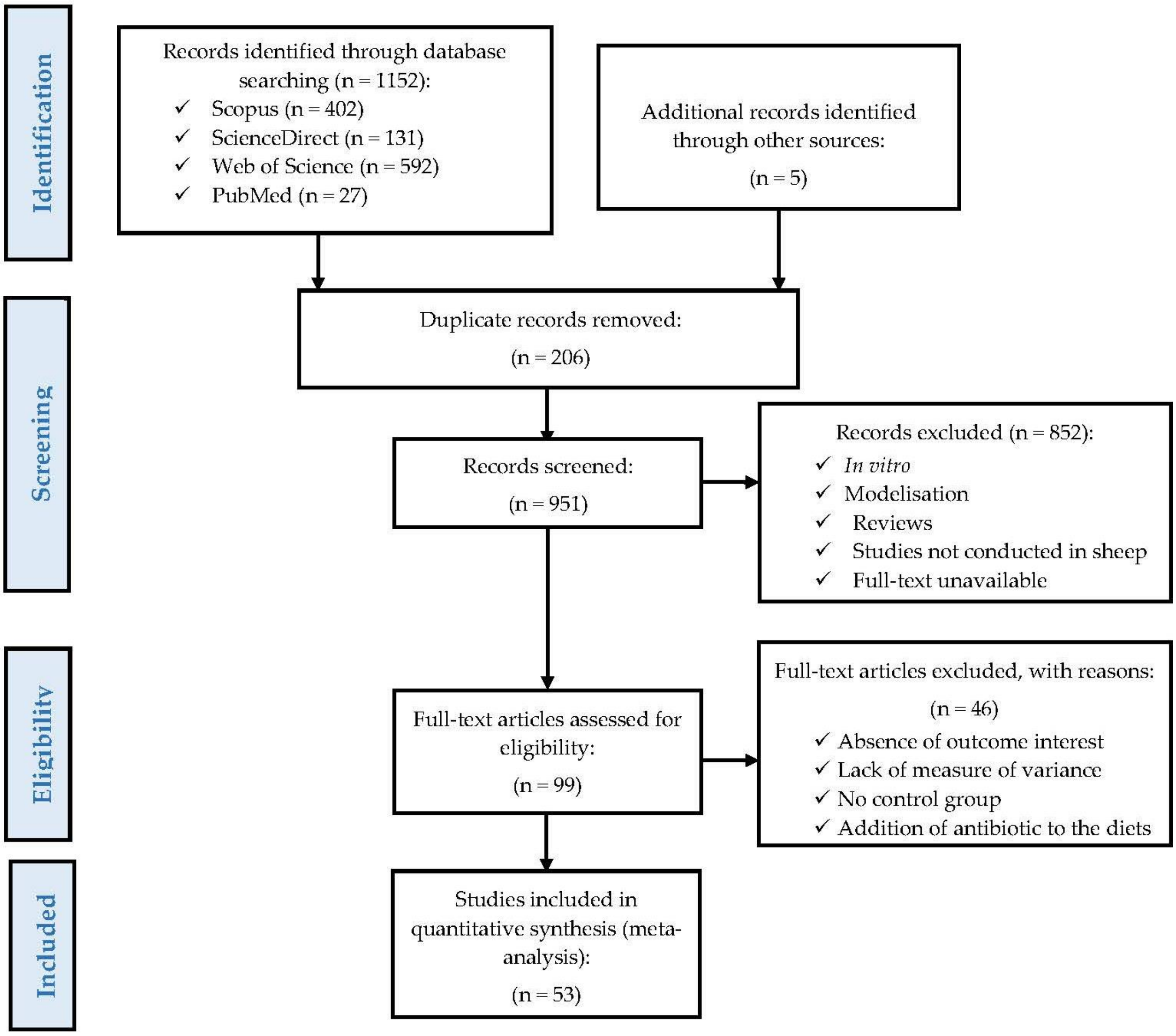

Figure S1. A PRISMA flow diagram detailing the literature search strategy and study selection for the meta-analysis.

Table S1. Subgroup analysis of the effect of dietary tannin dose in sheep.

			95 % CI			
**Subgroup**	**SMD**	**SE**	**Lower**	**Upper**	***p*-Value**	**N**
Daily weight gain (DWG)						
≤20 g/kg of DM	0.485	0.126	0.239	0.731	<0.001	70
>20 g/kg of DM	-0.282	0.247	-0.767	0.203	0.254	34
Dry matter intake (DMI)						
≤20 g/kg of DM	0.324	0.145	0.040	0.608	0.026	70
>20 g/kg of DM	-0.416	0.224	-0.856	0.023	0.063	34
Feed conversion ratio (FCR)						
≤20 g/kg of DM	-0.423	0.131	-0.679	-0.166	<0.001	53
>20 g/kg of DM	0.640	0.373	-0.090	1.370	0.086	7
Hot carcass yield (HCY)						
≤20 g/kg of DM	0.276	0.110	0.060	0.492	0.012	37
>20 g/kg of DM	0.152	0.228	-0.294	0.599	0.504	22
Cooking loss (CL)						
≤20 g/kg of DM	0.501	0.227	0.056	0.945	0.027	31
>20 g/kg of DM	-1.158	0.498	-2.133	-0.182	0.020	11
Intramuscular fat (IMF)						
≤20 g/kg of DM	0.207	0.264	-0.309	0.724	0.431	25
>20 g/kg of DM	-0.691	0.183	-1.050	-0.332	<0.001	15
Malondialdehyde in meat (MDAc)						
≤20 g/kg of DM	-2.735	0.458	-3.632	-1.838	<0.001	17
>20 g/kg of DM	-1.058	0.417	-1.875	-0.241	0.011	12

N: number of comparisons; SMD: standardized mean difference; CI: confidence interval of SMD; SE: standard error.

Table S2. Subgroup analysis of the effect of tannin supplementation period in sheep.

Subgroup	SMD	SE	95 % CI Lower	95 % CI Upper	*p*-Value	N
Daily weight gain (DWG)						
≤70 days	0.256	0.110	0.041	0.470	0.020	59
>70 days	0.515	0.233	0.058	0.972	0.027	41
Dry matter intake (DMI)						
≤70 days	-0.071	0.126	-0.318	0.177	0.576	59
>70 days	0.517	0.257	0.014	1.020	0.044	41
Feed conversion ratio (FCR)						
≤70 days	-0.197	0.147	-0.485	0.090	0.178	37
>70 days	-0.549	0.238	-1.016	-0.083	0.021	23
Redness (a*)						
≤70 days	-0.029	0.125	-0.273	0.215	0.816	31
>70 days	0.981	0.197	0.595	1.367	<0.001	23
Longissimus muscle area (LMA)						
≤70 days	0.063	0.168	-0.266	0.391	0.709	12
>70 days	0.870	0.284	0.313	1.427	0.002	10
Malondialdehyde in meat (MDAc)						
≤70 days	-0.784	0.233	-1.241	-0.327	<0.001	8
>70 days	-2.706	0.466	-3.620	-1.792	<0.001	21

N: number of comparisons; SMD: standardized mean difference; CI: confidence interval of SMD; SE: standard error.

Table S3. Subgroup analysis of the effect of age on the response to tannin supplementation in sheep.

Subgroup	SMD	SE	95 % CI Lower	95 % CI Upper	*p*-Value	N
Feed conversion ratio (FCR)						
≤3 months old	0.099	0.144	-0.184	0.381	0.494	23
>3 months old	-0.519	0.189	-0.890	-0.148	0.006	33
Longissimus muscle area (LMA)						
≤3 months old	-0.245	0.179	-0.596	0.106	0.171	6
>3 months old	1.108	0.322	0.477	1.738	<0.001	8
Redness (a*)						
≤3 months old	-0.061	0.148	-0.352	0.230	0.680	26
>3 months old	0.844	0.164	0.523	1.165	<0.001	27
Yellowness (b*)						
≤3 months old	-0.246	0.128	-0.497	0.005	0.055	26
>3 months old	0.502	0.272	-0.031	1.035	0.065	27
Intramuscular fat (IMF)						
≤3 months old	-0.498	0.182	-0.854	-0.141	0.006	20
>3 months old	0.553	0.367	-0.166	1.272	0.132	16
Malondialdehyde in meat (MDAc)						
≤3 months old	-0.320	0.173	-0.660	0.019	0.065	15
>3 months old	-4.489	0.607	-5.680	-3.298	<0.001	14

N: number of comparisons; SMD: standardized mean difference; CI: confidence interval of SMD; SE: standard error.

Table S4. Subgroup analysis of the effect of tannin type in sheep.

			95 % CI			
**Subgroup**	**SMD**	**SE**	**Lower**	**Upper**	***p*-Value**	**N**
Feed conversion ratio (FCR)						
Condensed tannins	-0.563	0.258	-1.069	-0.057	0.029	22
Hydrolysable tannins	-2.000	0.388	-2.670	-1.240	<0.001	2
Blend	-0.093	0.129	-0.346	0.161	0.474	36
Meat pH						
Condensed tannins	-0.111	0.140	-0.385	0.163	0.427	14
Hydrolysable tannins	-1.556	0.378	-2.297	-0.815	<0.001	2
Blend	0.199	0.117	-0.032	0.429	0.091	36
Lightness (L*)						
Condensed tannins	-0.072	0.156	-0.377	0.234	0.646	16
Hydrolysable tannins	1.373	0.690	0.020	2.725	0.047	4
Blend	-0.114	0.165	-0.438	0.210	0.489	34
Yellowness (b*)						
Condensed tannins	-0.500	0.137	-0.769	-0.232	<0.001	16
Hydrolysable tannins	3.312	1.568	0.239	6.384	0.035	4
Blend	0.123	0.158	-0.186	0.433	0.436	34
Malondialdehyde in meat (MDAc)						
Condensed tannins	-0.313	0.218	-0.739	0.114	0.151	10
Hydrolysable tannins	-0.106	0.334	-0.762	0.549	0.751	2
Blend	-3.666	0.543	-4.730	-2.602	<0.001	17

N: number of comparisons; SMD: standardized mean difference; CI: confidence interval of SMD; SE: standard error.

Table S5. Subgroup analysis of the effect of tannin inclusion method in sheep.

			95 % CI			
**Subgroup**	**SMD**	**SE**	**Lower**	**Upper**	***p*-Value**	**N**
Daily weight gain (DWG)						
Naturally present	0.422	0.133	0.161	0.683	0.002	82
Extract	-0.233	0.230	-0.685	0.218	0.311	22
Meat pH						
Naturally present	0.113	0.093	-0.070	0.296	0.228	47
Extract	-0.668	0.445	-1.540	0.203	0.133	5
Malondialdehyde in meat (MDAc)						
Naturally present	-2.664	0.413	-3.474	-1.855	<0.001	23
Extract	-0.159	0.219	-0.587	0.270	0.468	6

N: number of comparisons; SMD: standardized mean difference; CI: confidence interval of SMD; SE: standard error.

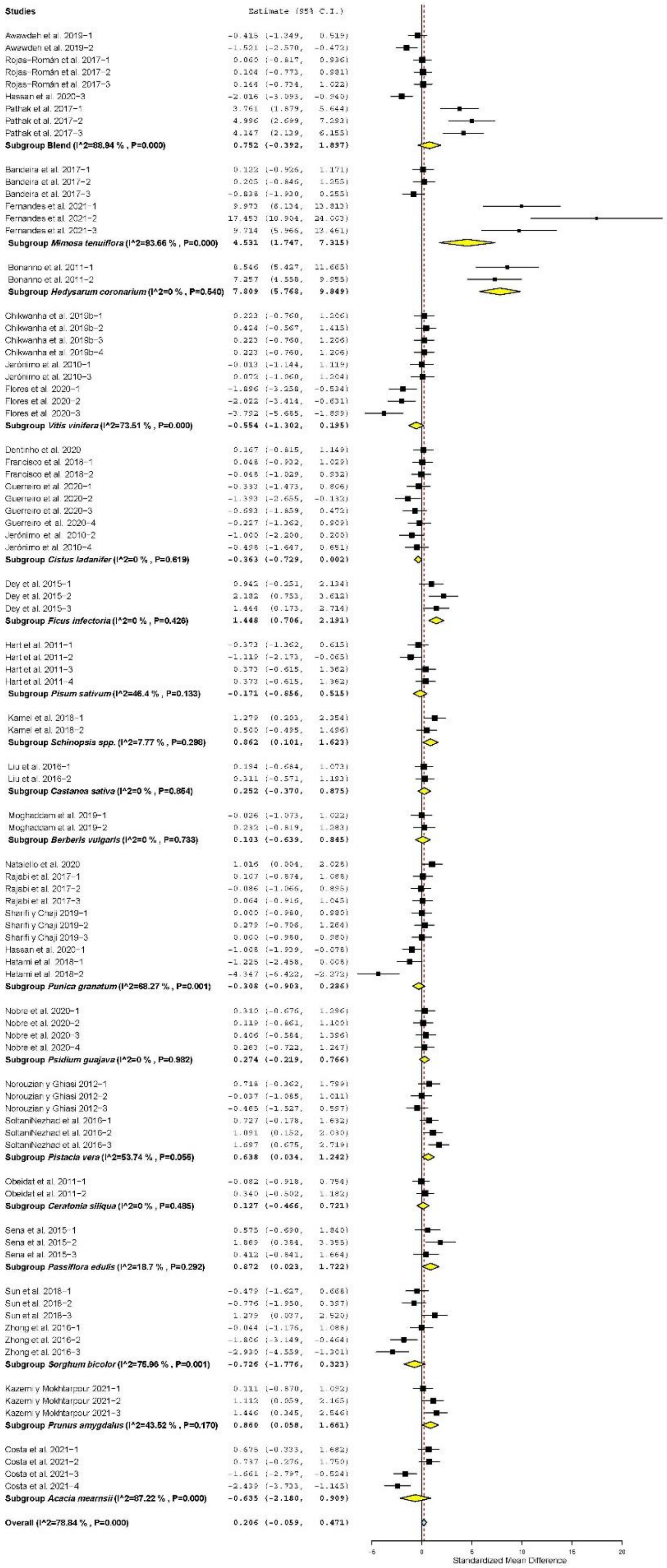

Figure S2. Forest plot of the effect size or standardized mean difference and 95% confidence interval of the source of botanical origin of tannin on dry matter intake (DMI) in sheep. The solid vertical black line represents the mean difference of zero or no effect. Points to the left of the solid vertical black line represent reduction in DMI, while points to the right of the line indicate increase in DMI.

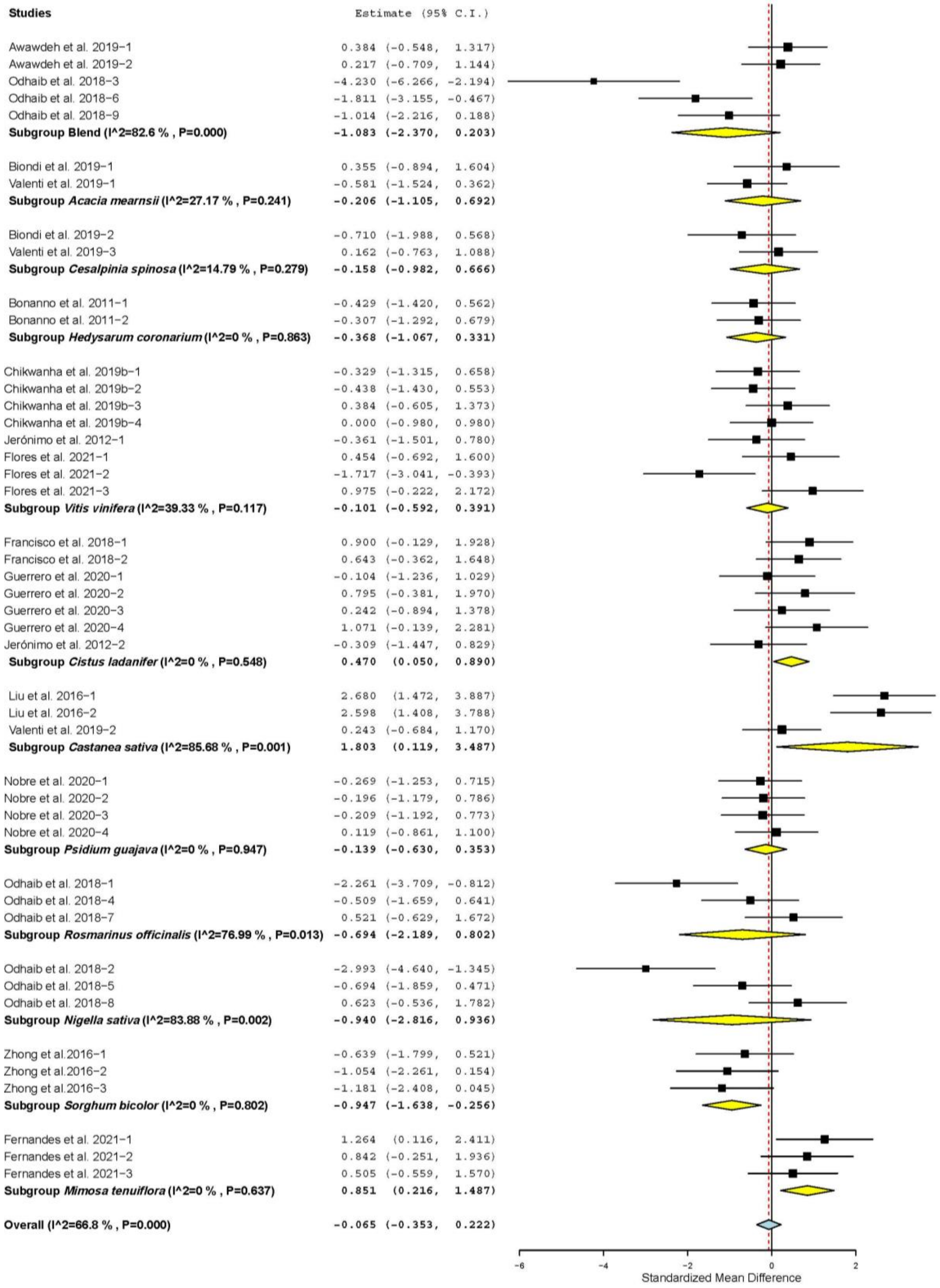

Figure S3. Forest plot of the effect size or standardized mean difference and 95% confidence interval of the source of botanical origin of tannin on the lightness (L*) of sheep meat. The solid vertical black line represents the mean difference of zero or no effect. Points to the left of the solid vertical black line represent reduction in L*, while points to the right of the line indicate increase in L*.

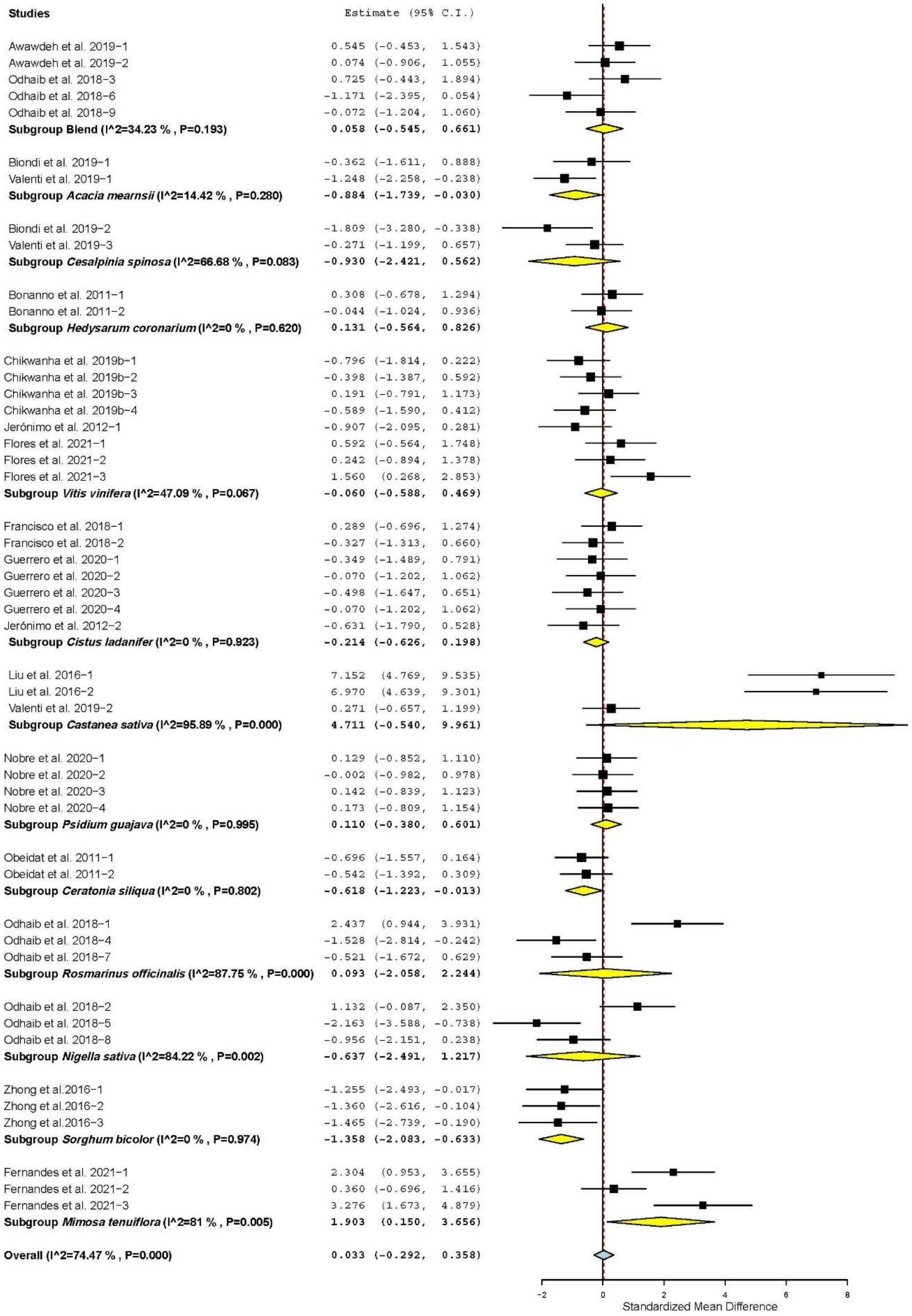

Figure S4. Forest plot of the effect size or standardized mean difference and 95% confidence interval of the source of botanical origin of tannin on the yellowness (b*) of sheep meat. The solid vertical black line represents the mean difference of zero or no effect. Points to the left of the solid vertical black line represent reduction in b*, while points to the right of the line indicate increase in b*.

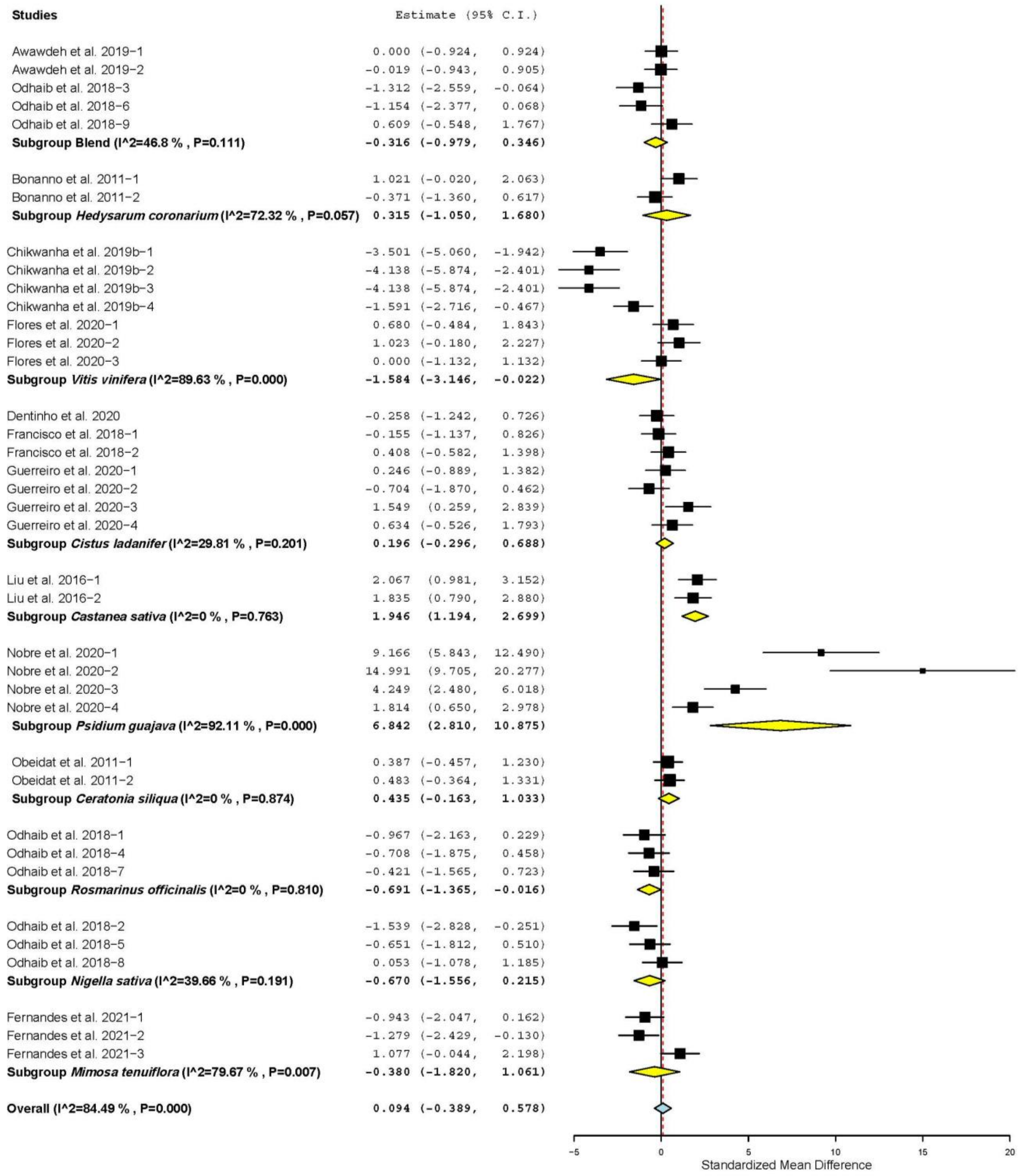

Figure S5. Forest plot of the effect size or standardized mean difference and 95% confidence interval of the source of botanical origin of tannin on cooking loss (CL) of sheep meat. The solid vertical black line represents the mean difference of zero or no effect. Points to the left of the solid vertical black line represent reduction in CL, while points to the right of the line indicate increase in CL.
